# Evaluation of cerebral hemodynamic assessment from time-of-flight magnetic resonance angiography using a topological network-based model with Transcranial Doppler measurements

**DOI:** 10.3389/fneur.2026.1805658

**Published:** 2026-07-02

**Authors:** Wheesung Lee, Sung-Ho Ahn, Do-Eun Lee, Tae-Rin Lee

**Affiliations:** 1 NEAR Brain Inc., Seoul, Republic of Korea; 2 Department of Neurology, Pusan National University Yangsan Hospital, Busan, Republic of Korea; 3 Research Institute for Convergence of Biomedical Science and Technology, Pusan National University School of Medicine, Busan, Republic of Korea

**Keywords:** cerebral hemodynamics, computational blood flow modeling, non-invasive cerebrovascular assessment, patient-specific hemodynamic modeling, time-of-flight magnetic resonance angiography

## Abstract

Quantitative measurement of cerebral hemodynamics is essential for the diagnosis, management, and prognostication of cerebrovascular diseases. Transcranial Doppler (TCD), though widely used for non-invasive blood flow velocity measurement, is inherently limited by inadequate acoustic windows and significant operator dependency and provides only localized velocity information without anatomical context. To address these limitations, Dr. NEAR flow (DNF) was developed to estimate key hemodynamic parameters, including blood flow rate, relative pressure, and velocity from routinely acquired time-of-flight magnetic resonance angiography (TOF-MRA). In this single-center retrospective study, the performance of DNF was evaluated in 113 patients by directly comparing DNF-calculated velocities with time-averaged mean velocities measured by TCD across seven major cerebral arteries. Patient-specific inflow boundary conditions were defined using TCD measurements to ensure physiologically consistent comparisons. Analysis of 700 arterial-level velocity pairs demonstrated a statistically significant positive correlation between the two modalities (Pearson’s correlation coefficient, *r* = 0.74, *p* < 0.0001). These results indicate that DNF offers a robust, non-invasive alternative for comprehensive cerebrovascular assessment, overcoming the procedural burdens and technical limitations associated with conventional TCD.

## 1 Introduction

Quantitative assessment of cerebral hemodynamics is crucial for the diagnosis, management, and prognostication of patients with cerebrovascular diseases, such as arterial stenosis, which can lead to ischemic stroke. By evaluating cerebral blood flow (CBF), clinicians can assess stroke severity and neurological status to predict patient outcomes within the critical early hours of onset, facilitating timely treatment decisions (1, 2). Despite this long-standing clinical need, it remains challenging to quantitatively measure blood flow velocity in complex cerebrovascular geometries without invasive procedures. For instance, transfemoral cerebral angiography remains a gold standard but requires post-procedural bed rest and carries risks of perioperative complications (3). Advanced MRI sequences, such as 4D flow MRI, provide non-invasive alternatives; however, while contrast agents are not always mandatory, they are often used to improve image quality, posing risks of hypersensitivity reactions, nephrotoxicity, and environmental impact (4, 5).

Currently, the most common non-invasive tool for hemodynamic assessment is Transcranial Doppler (TCD). As a portable and cost-effective technique, TCD allows for the dynamic measurement of beat-to-beat cerebral blood velocity (6). Consequently, it is widely employed in diagnosing various disorders, including acute ischemic stroke, vasospasm, and subarachnoid hemorrhage (7). However, TCD has inherent limitations that affect its clinical applicability, such as inadequate acoustic windows in a significant proportion of patients and a high degree of operator dependency (8). Furthermore, while TCD provides valuable flow information, it is difficult to use for absolute quantitative assessment of CBF because it relies solely on blood flow velocity measurements and necessitates simplified assumptions about vessel geometry (9, 10). Recent advances in the 3D reconstruction of intracranial arteries from neuroimaging have attempted to predict blood flow velocity and pressure using numerical methods. While these approaches have the potential to quantify hemodynamic information, they remain computationally intensive and impractical for routine bedside use (11, 12). Primary concerns include the difficulty of automatically generating relevant mesh structures in complex vascular geometries and the challenge of determining numerical parameters for patient-specific cases. As a result, TCD remains the de facto clinical reference for validating new computational tools (13, 14). Nevertheless, the inability of TCD to resolve vessel geometry or provide an integrated view of cerebral circulation underscores the need for innovative tools that can jointly capture vascular anatomy and functional hemodynamics.

To address these challenges, reduced-order modeling techniques— including one-dimensional (1D) vascular network and Poiseuille-based graph approaches—have been developed as computationally efficient alternatives to full 3D simulation (15, 16). Foundational 1D arterial blood flow formulations established reduced governing equations for pressure and flow wave propagation in compliant vascular networks (17, 18) and have since been progressively extended to anatomically detailed systemic arterial trees (19), to integrated systemic–cerebral models (16, 20), and to dedicated cerebral circulation models such as the Circle of Willis (21), with benchmark studies establishing cross-solver consistency for canonical test cases (22). In parallel, graph-based and resistance-network models have been used to compute pressure–flow relationships in realistic cerebral vascular networks and cortical microvascular beds (23, 24). By representing the vascular system as a network of connected segments, these methods deliver hemodynamic parameter estimates at a small fraction of the cost of full 3D simulation, making them particularly attractive for patient-specific assessment where rapid analysis and seamless integration with clinical imaging are essential. Because such models intentionally simplify vascular geometry and flow physics, their clinical accuracy hinges on two coupled ingredients: faithful patient-specific vessel reconstruction and physiologically appropriate boundary conditions, both of which must be substantiated through validation against *in vivo* measurements. The principal translational challenge therefore lies less in the modeling formulation itself than in establishing a robust workflow that integrates reduced-order hemodynamic analysis with routinely acquired clinical imaging.

Within this landscape, Dr. NEAR flow (DNF), the subject of this study, occupies the intersection of anatomically faithful 1D arterial network modeling and Poiseuille-based vascular graph analysis. DNF employs a 1D topological network-based computational technique to estimate key hemodynamic parameters—blood flow rate, relative pressure, and velocity—directly from routinely acquired time-of-flight magnetic resonance angiography (TOF-MRA), thereby grounding the network topology in patient-specific anatomy without invasive measurements or labor-intensive manual segmentation. Unlike TCD, which is limited to localized blood flow velocity measurements, DNF integrates patient-specific vascular geometry with functional hemodynamics to offer a comprehensive assessment of the cerebrovascular system. To ensure a consistent comparison with TCD’s localized measurements, TCD-measured velocity was used to define individualized inflow conditions for the DNF model. This approach ensures that both TCD and the DNF model operate under the same patient-specific flow context while leveraging MRA images to define unique vascular geometry. Validation was conducted through a direct comparison between the blood flow velocity predicted by DNF (*V_DNF_*) and the time-averaged mean velocity (TAMV) measured by TCD (*V_TCD_*). Demonstrating a strong concordance between these values would provide evidence that DNF can accurately capture individualized hemodynamics, laying the groundwork for its future application as a robust, non-invasive alternative to TCD.

## 2 Materials and methods

### 2.1 Patient selection

This single-center, retrospective study was approved by the Institutional Review Board (IRB No. 55–2024-044). All patients with stroke who were admitted to the Department of Neurology between September 2018 and October 2023 were screened. Final inclusion criteria were as follows: (i) available data from both TOF-MRA and TCD examinations, and (ii) a lesion involving the middle cerebral artery territory confirmed on imaging.

### 2.2 TOF-MRA data acquisition

High-resolution, three-dimensional brain TOF-MRA was performed for all included patients using a 3 T MRI scanner (Verio or Skyra; Siemens Healthineers, Erlangen, Germany). The MRA protocol utilized a 3D gradient-echo sequence with the following parameters: repetition time, 21 ms; echo time, 3.7 ms; flip angle, 18°; field of view, 167 × 260 mm^2^; slice thickness, 0.5 mm; and slice gap, 20 mm.

### 2.3 TCD data acquisition

TCD examinations were conducted by a single experienced neurosonographer using either a Pioneer TC 8080 (Nicolet Vascular, Madison, WI, USA) or Dolphin IQ (Viasonix, Netanya, Israel) system, both equipped with a 2-MHz pulsed-wave Doppler transducer. Major intracranial arteries were insonated through standardized acoustic windows: the occipital window for the vertebral, basilar, and posterior cerebral arteries; the orbital window for the internal carotid and anterior cerebral arteries; and the transtemporal window for the middle cerebral artery. For each artery, the following hemodynamic parameters were recorded: depth, peak systolic velocity, end-diastolic velocity, and TAMV. TAMV was specifically utilized as the core velocity variable for direct comparison with the DNF results and for the subsequent calculation of blood flow rate. Other derived indices, such as the pulsatility index, and resistive index, were also recorded.

### 2.4 Computation analysis using DNF

**Figure 1:**
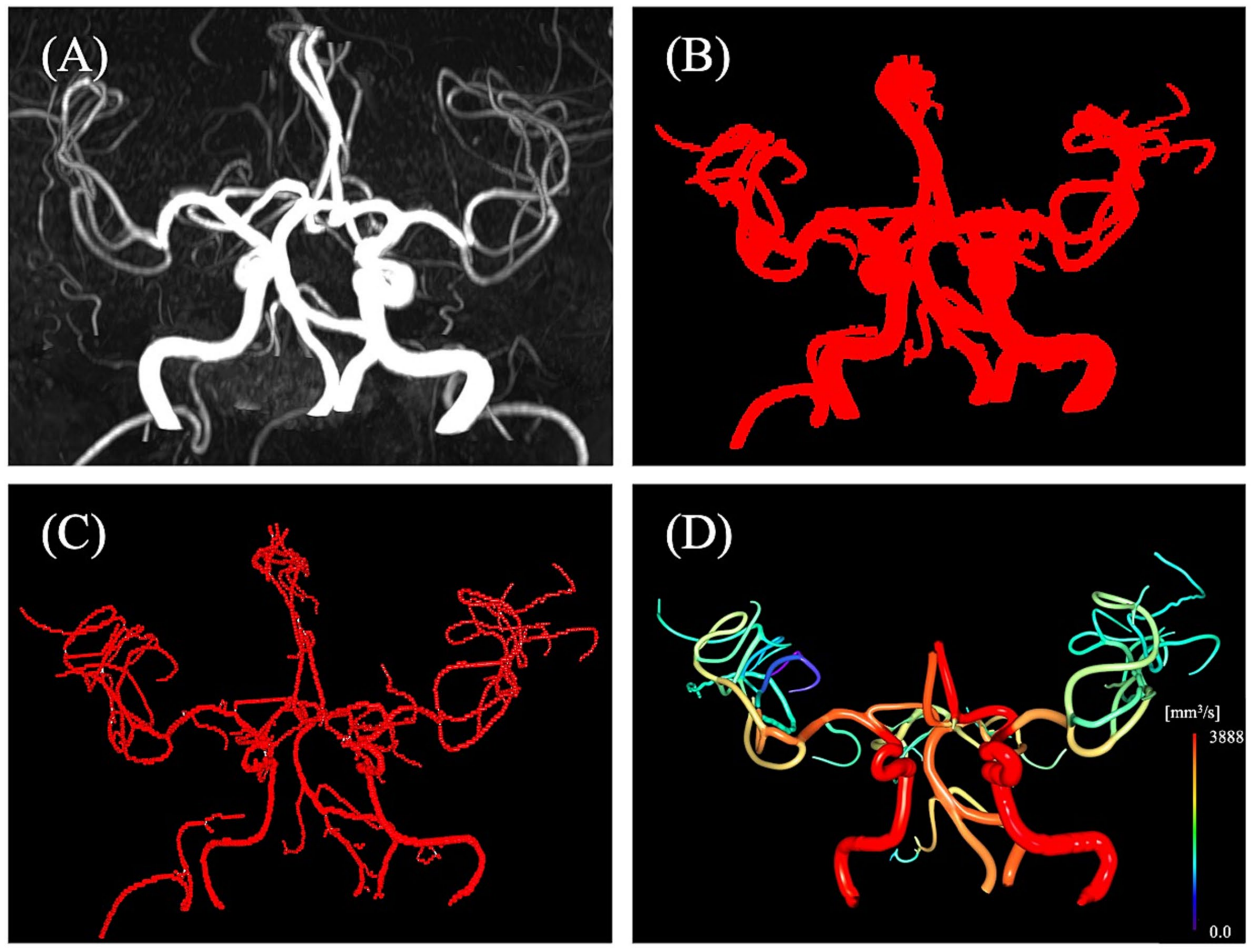
Step-by-step workflow of hemodynamic prediction using DNF. **(A)** Representative raw time-of-flight magnetic resonance angiography dataset. **(B)** Segmented three-dimensional vascular structure extracted from the MRA data after preprocessing and thresholding. **(C)** Simplified one-dimensional topological network model generated from the segmented vasculature, consisting of nodes and edges representing vessel connectivity. **(D)** Final visualization of the predicted hemodynamic parameters, including flow rate distribution across the vascular network. DNF, Dr. NEAR flow.

Computational blood flow analysis was performed using Dr. NEAR flow (NEAR Brain Inc., Korea). The analysis workflow, as summarized in Figure 1, comprised three major stages: vessel segmentation and refinement, skeletonization, and blood flow rate calculation. MRA-TOF images, provided in DICOM format, were imported into the software and preprocessed using Gaussian smoothing to reduce noise. Voxel extraction was performed via hysteresis thresholding to isolate the vascular structure. Coordinates of voxels exceeding the upper threshold were converted into a 3D point cloud, which was subsequently refined using Density-Based Spatial Clustering of Applications with Noise (DBSCAN) to eliminate outliers and non-vascular regions (25). Key parameters included threshold-based voxel selection and density-based clustering using DBSCAN, specifically the neighborhood radius parameter *ε* and the minimum number of points criterion (26). These settings were applied consistently across all cases to ensure stability and reproducibility of the segmentation process. To construct a graph representation of the vascular tree, each voxel was analyzed for topological connectivity with its neighbors. Voxels were classified as either branch points or intermediate nodes depending on their connectivity degree (27). A network graph structure was then generated by linking neighboring voxels that shared the same cluster label, forming a network of nodes and edges. The vascular network was represented as a patient-specific graph, with the number of nodes and vessel segments varying depending on individual vascular complexity. On average, the generated graphs consisted of several thousand nodes and segments per patient. Vessel radius was estimated by calculating the average Euclidean distance between each centerline node and its nearest vertices on the reconstructed 3D surface mesh. The final graph stored node identifiers, spatial coordinates, and estimated vessel radii, alongside connectivity information describing the vascular topology. The blood flow rate through each vessel segment was modeled based on the Poiseuille flow assumption, represented by the following equation:


$$Q=\frac{\pi {\bar{r}}^{4}}{8\mathrm{\eta L}}\;\mathrm{\Delta P}$$

where *Q* is the volumetric flow rate, $\bar{r}$ is the vessel radius, *η* is the blood viscosity, *L* is the vessel length, and Δ*P* is the pressure drop between the adjacent two nodes. Blood viscosity was assumed to be constant (***x*** = 4.265 × 10^−3^ Pa·s) (28). Based on the computed flow rates and radii, the blood flow velocity at each node was calculated by dividing the flow rate by the cross-sectional area, $A=\pi {\bar{r}}^{2}$ . In the Poiseuille-based network formulation, the vascular tree was discretized as a graph composed of interconnected nodes and vessel segments, with flow driven by pressure differences between adjacent nodes. Applying conservation of mass at each internal node yielded a system of linear equations describing pressure continuity and flow conservation throughout the network. The resulting sparse symmetric system was assembled in graph Laplacian form and solved under prescribed inflow and outlet boundary conditions to obtain the steady-state nodal pressure distribution. Because only relative pressure differences influence the computed flow distribution, pressure was calculated as a relative quantity within the vascular network. Accordingly, a fixed reference pressure of 10,000 Pa was imposed at the distal outlet nodes to close the system. After application of the boundary conditions, the resulting linear system became positive definite, enabling efficient computation of steady-state pressure, flow rate, and velocity distributions throughout the arterial network.

### 2.5 Boundary conditions and hemodynamic calculation

**Figure 2:**
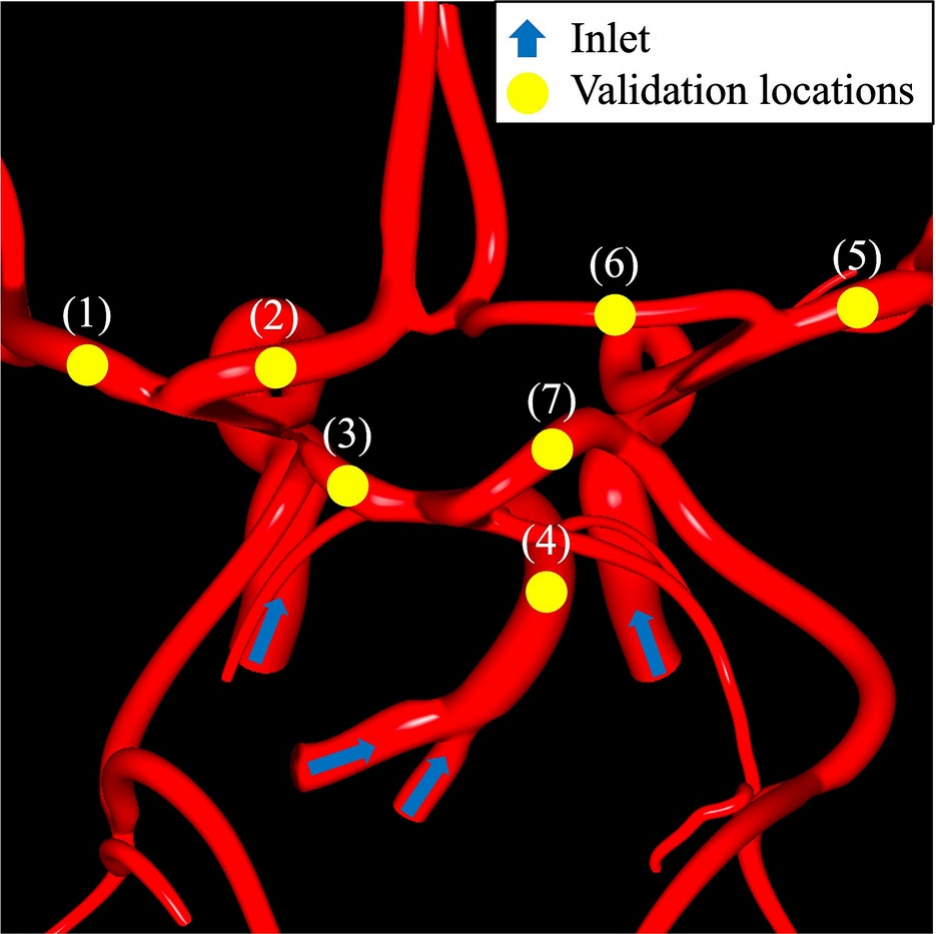
Schematic illustration of the cerebral arterial network used for validation. Blue arrows indicate inlet boundary conditions defined from Transcranial Doppler (TCD) measurements at proximal arteries. Yellow markers denote the locations where velocity measurements were compared between TCD and Dr. NEAR flow (DNF). The seven labeled arteries include: (1) left middle cerebral artery, (2) left anterior cerebral artery, (3) left posterior cerebral artery, (4) basilar artery, (5) right middle cerebral artery, (6) right anterior cerebral artery, and (7) right posterior cerebral artery.

Patient-specific inflow conditions were established using the TCD-measured TAMV. To determine inlet flow rates, the blood flow velocity measured at the internal carotid artery and vertebral artery (or basilar artery if vertebral artery data were missing) was multiplied by the corresponding vessel’s cross-sectional area, derived from the MRA-segmented radius. The locations of the inlet boundary conditions are illustrated in Figure 2. The resulting flow rates were prescribed at the inlets as Dirichlet boundary conditions, while pressure was specified at the distal outlets as a reference condition. This outlet condition defined the relative pressure field described above. By integrating patient-specific TCD data with MRA-derived geometry, the model ensures that hemodynamic predictions are grounded in the unique physiological context of each patient.

### 2.6 Statistical analysis

Statistical analyses were performed to compare blood flow velocity calculated by DNF with the corresponding TCD reference values. Comparisons were conducted in the basilar artery and bilaterally in the posterior cerebral artery, anterior cerebral artery and middle cerebral artery (Figure 2). The linear correlation between the two modalities was assessed using Pearson’s correlation coefficient, r. Furthermore, Bland–Altman analysis was employed to quantify the degree of agreement and identify any potential systematic biases or mean differences between the measurements. In addition, a linear mixed-effects model was applied with patient as a random effect to account for potential intra-patient correlation between arterial measurements obtained from the same individual.

## 3 Results

### 3.1 Clinical demographics and integrated DNF workflow

**Figure 3:**
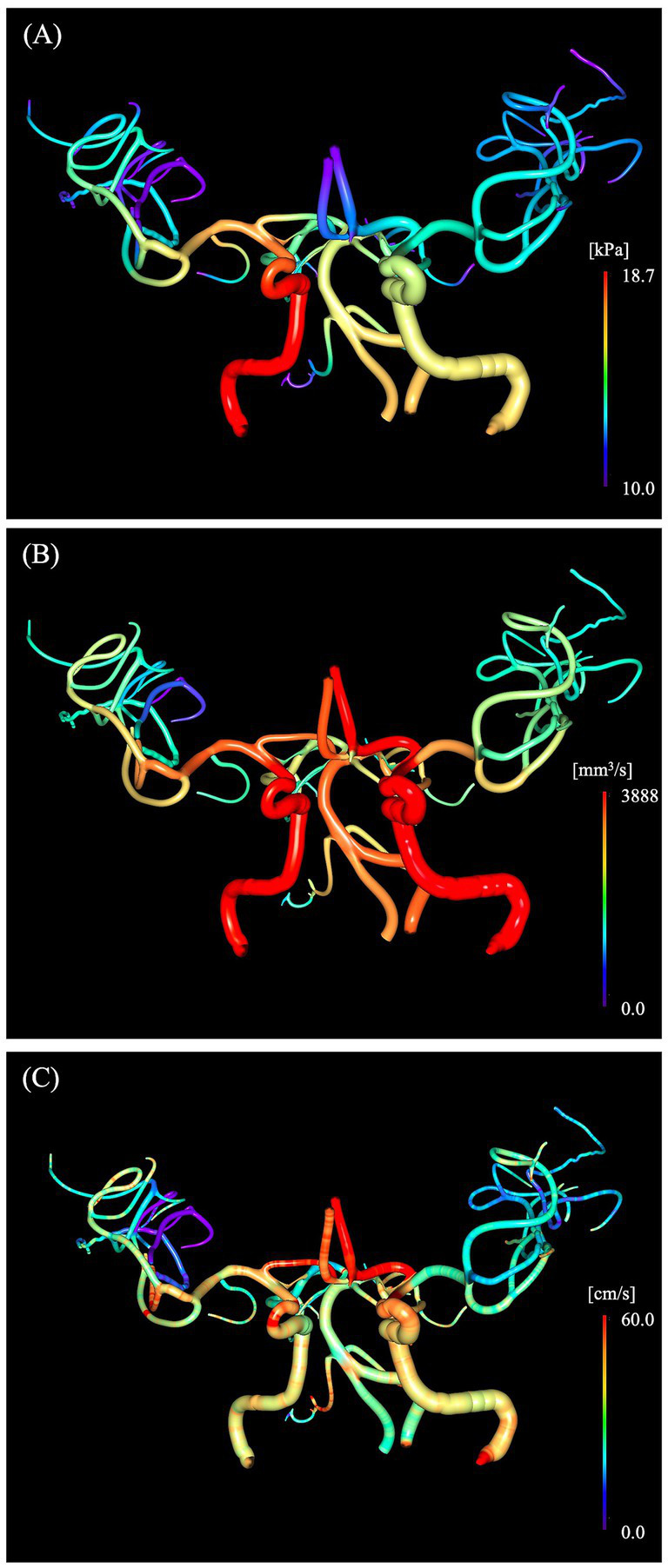
Visualization of Dr. NEAR flow (DNF)-calculated hemodynamic parameters in a representative cerebrovascular network. **(A)** Spatial distribution of blood flow rate across the vascular network. **(B)** Relative pressure distribution along the arterial tree. **(C)** Blood flow velocity distribution calculated from the model. All parameters were computed using patient-specific vascular geometry derived from time-of-flight magnetic resonance angiography.

DNF-based computational analysis was performed in all 113 patients included in the final analysis. The study cohort had a mean age of 67.23 ± 12.24 years, and 69.9% were male. Ischemic stroke was the primary diagnosis, accounting for 80.5% (*n* = 91) of cases. The mean interval between TCD and MRA scans was 6.52 ± 8.42 days (range: 0–63 days). Detailed demographic and clinical characteristics are summarized in Table 1. The DNF integrated workflow—comprising 3D vessel reconstruction from TOF-MRA and subsequent blood flow rate prediction—is illustrated in Figure 1. Patient-specific boundary conditions were primarily established at the internal carotid and vertebral arteries. In 31 cases where the bilateral vertebral arteries were not detectable on TOF-MRA, proximal input flow rate conditions were applied at the basilar artery inlet instead. Utilizing these datasets, DNF generated comprehensive hemodynamic profiles for each patient, including the spatial distribution of flow rates, relative pressure, and velocities throughout the cerebral arterial tree (Figure 3).

**Table 1:** Baseline characteristics of the study population.

Characteristic	Value
Age (years, mean ± SD)	67.23 ± 12.24
Sex, *n* (%)	
Male	79 (69.9)
Female	34 (30.1)
Clinical diagnosis, *n* (%)	
Ischemic stroke	91 (80.5)
Cerebral infarction	84 (74.3)
Cerebellar infarction	4 (3.5)
Pontine infarction	2 (1.8)
Thalamic infarction	1 (0.9)
Transient ischemic attack	5 (4.4)
Arterial stenosis	3 (2.7)
Other cerebrovascular conditions	14 (12.4)

*n*, number of subjects.

### 3.2 Global correlation and concordance between TCD and DNF

**Figure 4:**
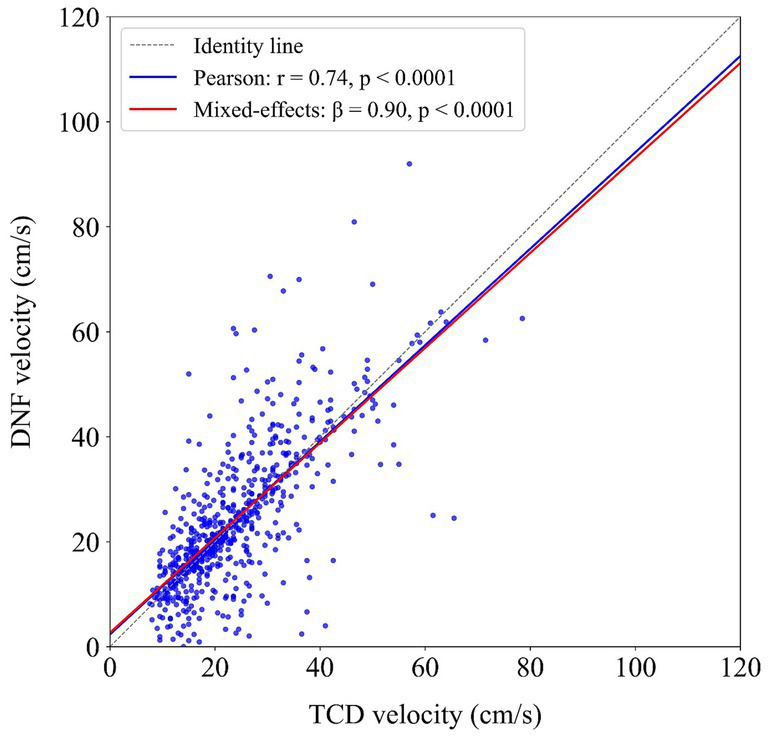
Scatter plot demonstrating the relationship between velocities measured using Transcranial Doppler (TCD) and those calculated using Dr. NEAR flow (DNF). The dashed gray line represents the identity line (y = x). The blue line indicates the Pearson correlation–based linear regression (*r* = 0.74, *p* < 0.0001), while the red line represents the linear mixed-effects model accounting for intra-patient clustering (*β* = 0.90, *p* < 0.0001).

**Figure 5:**
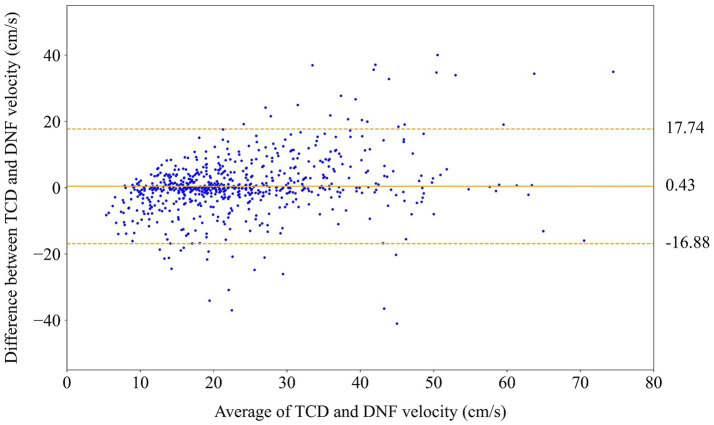
Bland–Altman plot assessing the agreement between the velocities measured with Transcranial Doppler (TCD) and those calculated with Dr. NEAR flow (DNF).

A total of 700 arterial-level velocity pairs were analysed across seven major cerebral arteries—the basilar artery and the bilateral posterior cerebral artery, anterior cerebral artery, and middle cerebral artery. *V_DNF_* demonstrated a statistically significant positive correlation with *V_TCD_* (Pearson’s correlation coefficient, r = 0.74, *p* < 0.0001) (Figure 4). To further account for potential intra-patient correlation, a linear mixed-effects model was applied with patient as a random effect. The association between TCD-measured and DNF-calculated velocities remained significant (*β* = 0.90, *p* < 0.0001), indicating that the observed relationship is robust even after accounting for clustering within patients (Figure 4). Concordance between the two modalities was observed in 94.3% of comparisons, indicating that DNF reliably captures the hemodynamic patterns detected by TCD across most of the cerebral vasculature. Bland–Altman analysis revealed a mean bias of 0.43 ± 8.83 cm/s (Figure 5). The observed bias suggests a minor systematic difference, with DNF tending to overestimate velocity.

Additional flow-based analysis further supported the consistency of the model. When TCD measurements were converted into flow rates using the MRA-derived cross-sectional areas at the insonation depth, the correlation between TCD-measured and DNF-calculated flow rates was higher (*r* = 0.88) than that observed for velocity measurements (*r* = 0.74). Nevertheless, the overall agreement between the two modalities confirms that DNF provides hemodynamically consistent measurements.

To evaluate the potential impact of temporal mismatch between MRA and TCD, a subgroup analysis was performed using a 7-day interval cutoff between the two examinations. Pearson’s correlation between TCD-measured and DNF-calculated velocities remained comparable in both groups (≤7 days: *r* = 0.74; >7 days: *r* = 0.76), suggesting that the time interval did not significantly influence the observed agreement.

### 3.3 Artery-specific hemodynamic agreement

To further evaluate the performance of DNF, artery-specific analyses were conducted (Table 2). The correlation between *V_TCD_* and *V_DNF_* ranged from moderate to strong across different vessels (Pearson’s correlation coefficient, r = 0.42–0.80, all *p* < 0.0001). While mean velocity values were generally comparable, DNF tended to yield slightly higher velocity estimates than TCD. The sustained correlation strength across all examined vessels reinforces that DNF is a robust tool for hemodynamic assessment throughout the cerebral arterial network.

**Table 2:** Artery-specific comparison between TCD (*V_TCD_*) and DNF calculated velocity (*V_DNF_*).

Artery	*n*	*V_TCD_* (cm/s)	*V_DNF_* (cm/s)	*r*	*p*
BA	81	19.22 ± 7.97	21.58 ± 8.41	0.80	< 0.0001
PCA-L	102	15.41 ± 4.90	15.39 ± 7.61	0.42	< 0.0001
PCA-R	103	15.52 ± 4.10	15.40 ± 8.08	0.62	< 0.0001
ACA-L	97	26.38 ± 8.95	26.63 ± 13.95	0.66	< 0.0001
ACA-R	103	26.05 ± 8.35	27.07 ± 14.23	0.57	< 0.0001
MCA-L	107	30.08 ± 10.26	29.47 ± 11.61	0.68	< 0.0001
MCA-R	107	30.04 ± 13.42	30.59 ± 15.13	0.78	< 0.0001

TCD, Transcranial Doppler; DNF, Dr. NEAR flow; n, number of arteries; r, Pearson’s correlation coefficient; p, statistical significance; BA, basilar artery; PCA, posterior cerebral artery; ACA, anterior cerebral artery; MCA, middle cerebral artery; L, left; R, right.

## 4 Discussion

This study demonstrates that DNF can reliably estimate cerebral hemodynamic parameters using routinely acquired TOF-MRA data, enabling flow quantification without the need for modified protocols, additional imaging, or contrast agents. The finding of a strong correlation and agreement between *V_TCD_* and *V_DNF_* is a critical first step in validating this technology however, the current validation is not fully independent because TCD-measured velocities were used to define the proximal inflow boundary conditions for the DNF simulations. Consequently, the observed agreement may partly reflect incorporation of TCD information into the computational model rather than a completely independent comparison between modalities. TCD has several limitations, including the absence of adequate acoustic windows in up to 15% of patients over age 60 and significant inter-operator variability (8). In addition, velocity measurements are inherently dependent on the insonation angle, as the measured velocity represents the projection of true flow along the ultrasound beam direction. Deviations from optimal alignment lead to systematic underestimation of the true velocity due to the cosine relationship between measured and actual flow velocity (29, 30). Despite these limitations, TCD remains the de facto clinical reference for hemodynamic monitoring. Therefore, demonstrating that DNF aligns with this established standard is essential for establishing its clinical relevance. Importantly, the TCD-derived inputs were restricted to proximal inflow conditions, whereas the downstream distribution of flow rate, pressure, and velocity across the cerebrovascular network was governed by the reconstructed vascular geometry and physical flow laws independent of direct TCD measurements. Therefore, the observed agreement across the cerebrovascular network suggests that the model can reproduce physiologically plausible patient-specific hemodynamic patterns beyond the prescribed inflow parameters. Future studies should investigate the robustness of the model under alternative inflow conditions independent of TCD measurements, such as boundary conditions derived from independent reference modalities or systemic physiological parameters including heart rate and blood pressure. Such approaches would enable a more rigorous independent validation of the proposed framework while further assessing its ability to reproduce patient-specific cerebrovascular hemodynamics.

From a clinical perspective, these findings suggest that DNF has the potential to complement existing hemodynamic assessment tools by providing a more comprehensive evaluation of the cerebrovascular system. Unlike TCD, which is limited to localized velocity measurements, DNF enables simultaneous multi-vessel assessment and provides additional parameters such as flow rate and relative pressure across the vascular network. By extracting key parameters, such as vessel centerlines and radii, from 3D vascular geometry, DNF enables a consistent and objective evaluation of various metrics, including blood flow rates and pressure distributions throughout the intracranial arterial system. This capability may be particularly valuable in patients with limited acoustic windows or in clinical scenarios requiring global hemodynamic assessment, such as multi-vessel disease. Taken together, these features suggest that DNF may extend the current paradigm of cerebrovascular evaluation beyond point-based measurements toward a more integrated anatomical and functional assessment, potentially facilitating comprehensive hemodynamic evaluation in routine clinical settings, although further studies are required to establish its direct impact on clinical decision-making and patient outcomes.

Although the DNF model demonstrated promising capability in approximating cerebrovascular hemodynamics, several model-related limitations concerning the simplification of hemodynamic characteristics should be acknowledged. The DNF model treats blood as a Newtonian fluid with constant viscosity, which does not fully capture complex rheological properties such as viscoelasticity and shear-thinning behavior (31, 32). In physiological arterial flow, secondary patterns arising from vessel curvature, bifurcations, or changes in cross-sectional area significantly influence local hemodynamics. Newtonian models may fail to adequately represent the interaction between these geometrically induced flow complexities and non-linear rheological properties, potentially affecting the predicted velocity even within large vessels (32). Although Newtonian and non-Newtonian models tend to converge at high shear rates (≥100 s^−1^), physiological shear rates vary widely throughout the cardiac cycle (0–1,000 s^−1^). The omission of these effects likely introduces discrepancies in velocity estimation, particularly during periods of low shear; thus, incorporating viscoelastic characteristics in future iterations would enhance the model’s physiological fidelity (32–34). In addition, the current model assumes steady flow conditions based on time-averaged velocities, which do not fully capture the pulsatile nature of cerebral blood flow. Temporal variations in velocity and pressure throughout the cardiac cycle may influence local hemodynamics, and the use of a steady-flow approximation may contribute to discrepancies between predicted and measured values. Furthermore, the model relies on vessel radius derived from TOF-MRA, which introduces an important source of uncertainty. Under the Poiseuille formulation, flow rate is proportional to the fourth power of vessel radius, meaning that even small errors in radius estimation can lead to substantial deviations in calculated flow rate and velocity. This sensitivity may be particularly pronounced in smaller vessels or in regions where image resolution and signal loss limit accurate vessel delineation. Despite these limitations, the model remains a reasonable approximation for large cerebral arteries, where flow is predominantly laminar and shear rates are relatively high. Therefore, while local discrepancies may occur, particularly in regions of complex flow or small vessel caliber, the model is expected to reliably capture overall hemodynamic patterns across the cerebrovascular network.

Bland–Altman analysis in Figure 5 revealed that DNF tends to slightly overestimate velocity relative to TCD. This systematic bias may be attributed to the inherent limitations of TOF-MRA imaging, such as image noise, signal loss in regions of turbulent or in-plane flow, and the under-representation of small vessels due to saturation effects (35). These artifacts can result in incomplete or distorted reconstruction of the vascular geometry, thereby influencing vessel radius estimation and the resulting hemodynamic calculations. The coexistence of strong correlation and non-negligible limits of agreement suggests that DNF more reliably captures relative hemodynamic patterns across the cerebrovascular network than precise point-specific absolute velocity values. Interpretation of Bland–Altman limits of agreement should also be considered in the context of the intended clinical application, as acceptable agreement ranges are application-specific and ideally should be defined *a priori* according to clinical decision requirements (36). To our knowledge, clinically meaningful limits of agreement for noninvasive cerebrovascular hemodynamic modeling remain insufficiently defined. Therefore, while the present findings support the overall hemodynamic consistency of the DNF framework, further studies are needed to establish clinically meaningful agreement thresholds for specific diagnostic and prognostic applications.

**Figure 6:**
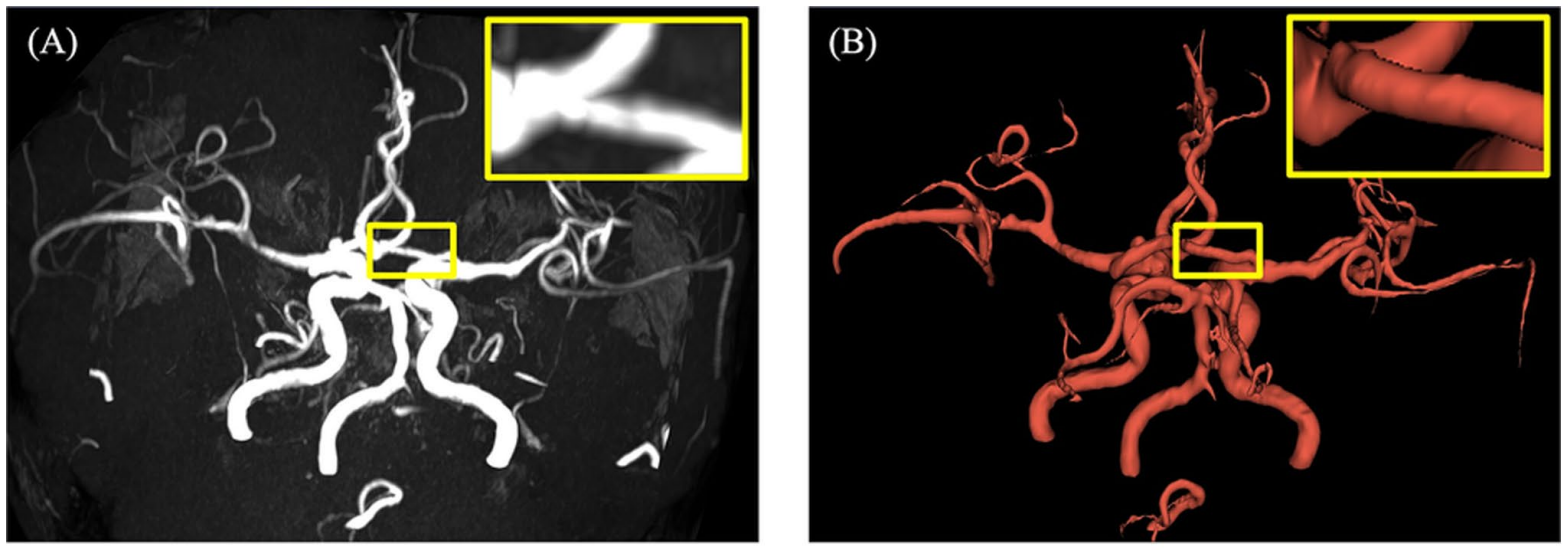
Representative case demonstrating the impact of MRA reconstruction quality on the agreement between Transcranial Doppler (TCD) and Dr. NEAR flow (DNF). **(A)** Original MRA maximum intensity projection image showing heterogeneous signal intensity in the right anterior cerebral artery. **(B)** Corresponding DNF-derived three-dimensional vascular reconstruction.

Despite the strong overall correlation, Figure 4 demonstrates increasing discrepancies between DNF and TCD at higher velocity ranges, which may be attributed to both measurement- and modeling-related factors. First, TCD velocities are sensitive to the insonation angle, and small deviations from optimal alignment can lead to larger absolute errors at higher velocities. In addition, operator-dependent factors such as probe positioning may further increase variability, particularly in regions where velocity gradients are steep, and small spatial differences can result in substantial changes in measured values. From a modeling perspective, uncertainties in boundary conditions derived from TCD measurements may further influence the predicted hemodynamic parameters. As inflow conditions are based on time-averaged velocities, measurement variability or angle-dependent error in TCD may propagate through the computational model and affect the resulting hemodynamic patterns. Representative case further demonstrated that agreement between TCD and DNF may vary depending on the quality of vascular reconstruction from TOF-MRA (Figure 6). In regions with preserved vessel continuity and clear delineation of the arterial lumen, DNF-calculated velocities showed close agreement with TCD measurements. In contrast, larger discrepancies were observed in arterial segments affected by heterogeneous signal intensity. In the representative case shown in Figure 6, most arterial segments demonstrated relatively small velocity differences between TCD and DNF (absolute differences: 0.77–3.36 cm/s), whereas the right anterior cerebral artery showed a substantially larger discrepancy, with the DNF-calculated velocity exceeding the TCD-measured velocity by 16.83 cm/s. Notably, this segment demonstrated non-uniform signal intensity on TOF-MRA and appeared artificially enlarged in the reconstructed vascular model, suggesting that segmentation-related uncertainty in vessel radius estimation contributed to the observed discrepancy. These findings further support that image quality and segmentation accuracy are important determinants of model performance, particularly given the strong sensitivity of Poiseuille-based flow estimation to vessel radius.

Further validation against established flow measurement modalities is needed to confirm technical reliability and address potential modeling limitations of DNF. Non-invasive techniques such as phase-contrast MRI and 4D flow MRI provide promising alternatives for quantitative flow measurement and may serve as valuable reference standards for future validation studies. A direct comparison between parameters obtained from DNF and those measured using these modalities would further strengthen the technical reliability of the model. Additionally, while cerebral angiography remains the gold standard for anatomic imaging, its invasive nature, requirement for contrast agents, and associated procedural risks make it unsuitable for routine hemodynamic assessment (37, 38). Nevertheless, a future study directly comparing DNF-calculated values with flow rates measured during invasive angiography would be a significant step in validating the model’s technical accuracy. Furthermore, future research should investigate whether DNF-calculated parameters correlate with clinical outcomes to determine their prognostic significance and broader clinical utility.

## Data Availability

The datasets presented in this article are not readily available because patient privacy and institutional review board regulations. Requests to access the datasets should be directed to Tae-Rin Lee, taerin@nearbrain.com and Sung-Ho Ahn, caesar-ahn@hanmail.net.
